# Origin and Evolution of RNA-Dependent RNA Polymerase

**DOI:** 10.3389/fgene.2017.00125

**Published:** 2017-09-20

**Authors:** Savio T. de Farias, Ariosvaldo P. dos Santos Junior, Thais G. Rêgo, Marco V. José

**Affiliations:** ^1^Laboratório de Genética Evolutiva Paulo Leminsk, Departamento de Biologia Molecular, Universidade Federal da Paraíba João Pessoa, Brazil; ^2^Departamento de Informática, Universidade Federal da Paraíba João Pessoa, Brazil; ^3^Theoretical Biology Group, Instituto de Investigaciones Biomédicas, Universidad Nacional Autónoma de México Mexico City, Mexico

**Keywords:** origin of life, virus evolution, RNA world, tRNAs, RdRp

## Abstract

RNA-dependent RNA polymerases (RdRp) are very ancient enzymes and are essential for all viruses with RNA genomes. We reconstruct the origin and evolution of this polymerase since the initial stages of the origin of life. The origin of the RdRp was traced back from tRNA ancestors. At the origin of the RdRp the most ancient part of the protein is the cofactor-binding site that had the capacity of binding to simple molecules as magnesium, calcium, and ribonucleotides. Our results suggest that RdRp originated from junctions of proto-tRNAs that worked as the first genes at the emergence of the primitive translation system, where the RNA was the informational molecule. The initial domain, worked as a building block for the emergence of the fingers and thumb domains. From the ancestral RdRp, we could establish the evolutionary stages of viral evolution from a rooted ancestor to modern viruses. It was observed that the selective pressure under the RdRp was the organization and functioning of the genome, where RNA double-stranded and RNA single-stranded virus formed a separate group. We propose an evolutionary route to the polymerases and the results suggest an ancient scenario for the origin of RNA viruses.

## Introduction

RNA-dependent RNA polymerases (RdRp) are essential enzymes for all viruses with RNA genomes (Černý et al., 2014). Unlike other polymerases, the RdRp have high mutation rates (in the order of 10^-4^), which is crucial to generate the variability of RNA viruses, being important to their evolutionary strategy (Vignuzzi et al., 2006). These enzymes also participate in several biological processes in eukaryotes, such as amplification of microRNAs (Alquist, 2002), which are involved in the control of the genetic expression, on the development of nematodes, and in protecting plants against pathogenic agents (Iyer et al., 2003; Zong et al., 2009). The 3D structural conformation of RdRp displays a right hand with three functional subdomains, called fingers, palm, and thumb. In all polymerases, the palm subdomain is highly conserved. In RdRp, the palm subdomain has two conserved residues of aspartate, which interacts with two divalent metal ions that facilitate a nucleophilic attack, where this interaction allows the incorporation of the incoming ribonucleotide of the chain into formation. At the fingers subdomain occurs the interaction with the template and with the nucleotide during the polymerization. In the N-terminal portion of the enzyme is located the thumb subdomain, that is the most variable part of the protein (Mönttinen et al., 2014; Jácome et al., 2015). In all RdRp there are six conserved structural motifs (A–F), located in its majority in the palm subdomain (A–E motifs) and the F motif is located on the finger subdomain. All these motifs have been shown to be related with processes of correct incorporation and reorganization of nucleotides. Several of these motifs are shared with other polymerases, indicating their fundamental importance in the enzymatic function. These motifs are in proximity to the catalytic site that is responsible for the binding of substrates and cofactors (Oreilly and Kao, 1998; Ng et al., 2008). With the discovery of the catalytic function of RNA molecules (Kruger et al., 1982; Guerrier-Takada et al., 1983), it was suggested that RNA had the dual role of storing biological information in their sequences, and they also acted as catalysts, which were crucial for the biological systems in formation (Gilbert, 1986). Another hypothesis, based in structural phylogenomic reconstruction, suggests a coevolution between nucleic acids and proteins that participated in the primordial organization of the primitive translation system (Caetano-Anollés and Seufferheld, 2013). In both hypothesis, with the synthesis of the first proteins, the RdRp may have played a pivotal role in early evolution, when RNA was the genetic material. The emergence of these enzymes made possible the replication and perpetuation of the information stored in this initial genome (Müller, 2006). Several authors have suggested a similar origin between tRNA and rRNA or mRNA (Bloch et al., 1984, 1989; Tamura, 2011; Root-Bernstein and Root-Bernstein, 2015, 2016). Farias et al. (2014, 2016a) suggested a new model for the origin of the biological systems, where the tRNA molecules orchestrated the initial organization of life. According to the tRNA core hypothesis (Farias et al., 2016a), the tRNA worked as the primitive genetic material, and gave rise to mRNA and rRNA, as well as, alternative structural conformations of the first ribozymes. With the emergence of the rRNA and mRNA, this system could originate the primitive translation system, which made possible the synthesis of the first proteins (Jheeta, 2015; Farias et al., 2016a). The origin of the primitive translation system allowed the transition from an RNA world to an RNA/protein world, and subsequently to DNA/RNA/protein world (Farias et al., 2014, 2016a). Farias et al. (2016b) suggested the composition of the proteome before the last universal common ancestor based on the translation of tRNA ancestor molecules. Among the modern proteins that had similarities with translated tRNA ancestor sequences, the RdRp was the only polymerase that had a match (Farias et al., 2016b). This result reinforces the nature of the primitive genetic material based in RNA and the early emergence of the RdRp in the biological systems.

Herein, we used the sequences derived from RNA ancestor molecules that when translated had matches with the RdRp, as suggested by Farias et al. (2016b), to reconstruct the origin and evolution of this polymerase in the early stages of the biological systems. We also describe the evolutionary stages of viral evolution.

## Materials and Methods

### Reconstruction of the Ancestral Structure and Binding Analysis

Farias (2013) reconstructed the ancestral sequences of tRNAs. From these ancestral sequences, Farias et al. (2016b) combined eight ancestral sequences in all permutations for the amino acids (Ile, Asn, Vau, Asp, Gly, Ser, Thr, and Ala), translated and compared them with the modern proteins. These amino acids were suggested by Eigen and Schuster (1977) as the first amino acids that were incorporated in the primeval genetic code. Farias et al. (2016b) suggested that the RdRp emerged from junctions of ancestral tRNAs. Four ancestral sequences of RdRp were used in this work as suggested by Farias et al. (2016b). Each ancestral sequence was submitted to the web server I-Tasser (Zhang, 2008)^1^ to predict by homology the best structural model. For a better structural resolution of the ancestral proteins, each structural ancestral model was submitted to the program ModRefiner (Xu and Zhang, 2011)^2^, which refined all the stems giving a better resolution for each ancestral model. After refinement, each structural model was submitted to the program COACH (Yang et al., 2013)^3^ to infer which molecule best binds to the binding site of the ancestral proteins. In all programs, the default parameters were used.

### Structural Comparative Analysis

For the comparative analysis between the ancestral structural models and the modern proteins the following crystallized structures for RdRp were obtained from the Protein Database: RNA Polymerase DSRNA Bacteriophage (PDB: 1HHS); RNA Polymerase Rabbit Hemorrhagic Disease Virus (PDB: 1KHV); RNA Polymerase Sapporo Virus (PDB: 2CKW); Hepatitis C RNA Polymerase (PDB: 2D41); Neurospora Crassa RNA Polymerase (PDB: 2J7N); RNA Polymerase Birnavirus (PDB: 2PGG); RNA Polymerase Infectious Bursal Disease Virus (PDB: 2PUS); RNA Polymerase Rotavirus (PDB: 2R7T); RNA Polymerase Infectious Pancreatic Necrosis Virus (PDB: 2YI8); RNA Polymerase Cypoviruses (PDB: 3JA4); Enterovirus A RNA Polymerase (PDB: 3N6L); RNA Polymerase Norwalk Virus (PDB: 3UQS); RNA Polymerase Rotavirus A (PDB: 4AU6); RNA Polymerase Thosea Assigns Virus (PDB: 4XHA); Rhinovirus A (PDB: 1XR7); Enterovirus C (PDB: 3OL6); Foot-and-Mouth Disease Virus (PDB: 1U09); Cardiovirus A (PDB: 4NZ0); Japanese Encephalitis Virus (PDB: 4HDH); Dengue Virus (PDB: 2J7U); Bovine Viral Diarrhea Virus 1 (PDB: 1S48); Qbeta Virus (PDB: 3MMP); Reovirus (PDB: 1MUK); and La Crosse Bunyavirus (PDB: 5AMQ). In Supplementary Table S1 (Supplementary Information), the RMSD values of the structural alignment between the tRNA genes ancestors and modern proteins are shown. From the structures of RpRd, obtained from the Protein Data Bank, the ancestral sequence was calculated by maximum likelihood and the structure was inferred using the I-TASSER web server. We used the ancestral sequence and structure as a control for the structures obtained from the translation of tRNA ancestor sequences. For the structural alignments, the program TM-align (Zhang and Skolnick, 2005)^4^ was used. All modern proteins were aligned with the four ancestral structures and the control. The RMSD value was calculated for each structural alignment. The RMSD values were plotted in a matrix and the NJ tree of the structure was computed in the T-Rex web server (Boc et al., 2012)^5^.

## Results

**FIGURE 1:**
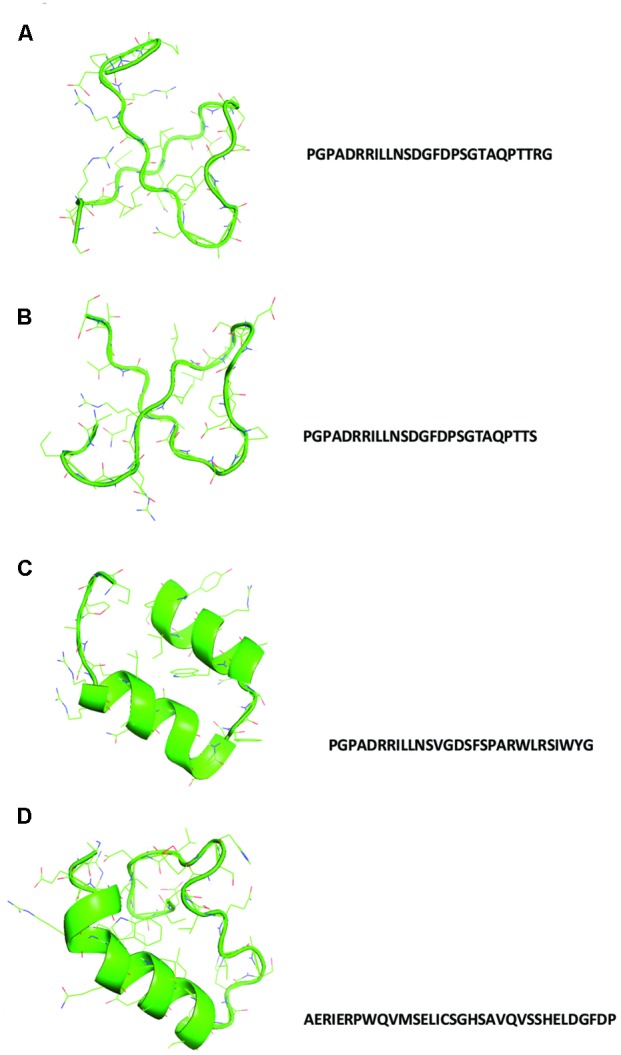
Structural models with their respective sequences generated using the translated proto-tRNAs concatamers as suggested by Farias et al. (2016b). **(A)** tRNA 1 model, **(B)** tRNA 2 model, **(C)** tRNA 3 model, and **(D)** tRNA 4 model.

**FIGURE 2:**
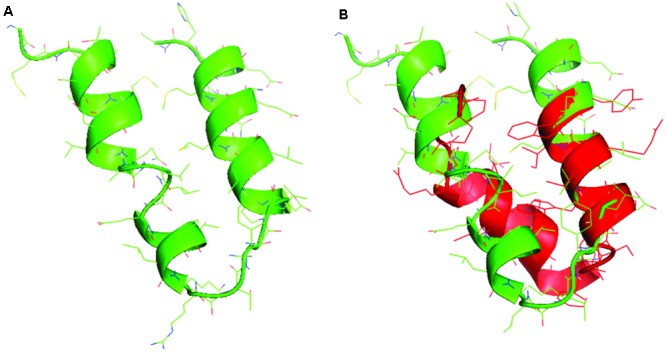
**(A)** The structural model for the control and **(B)** the structural alignment between the control (green) and the tRNA 3 model (red).

In **Figure 1**, the sequences and the structural models generated by homology to ancestral sequences, as suggested by Farias et al. (2016b), are shown. The obtained structures show a simple conformation with loops and helices and the sequences proposed in **Figure 1A** tRNA1, **Figure 1B** tRNA 2, and **Figure 1C** tRNA 3 showed similarities among them, with small differences in the amino acids composition. The sequence in **Figure 1D** tRNA 4 is the most divergent and can represent a second module in the formation of the primordial protein. Alva et al. (2015) proposed the same structural domains as an ancient vocabulary of peptides used in the evolutionary process to give origin to the diversity of structural folding in modern proteins. Aziz et al. (2016) analyzed the functional early motifs and domains, suggesting that loop motifs and domains structures worked as connection points for the recruitment and complexification of the initial protein networks. In **Figure 2A**, note the structure of the ancestral control based in the three dimensional structure of proteins as obtained from the Protein Data Bank, and in **Figure 2B**, the structural alignment between the control and the tRNA3. The RMSD value of the structural alignment was 2.75, despite the high value of the structural conformation, indicating that either the tRNA 3 and the control can have the same ancestor or that they are at different points of the same evolutionary history. The results suggest that the ancestral sequence and structure are convergent, which in turn indicates that the origin of the RdRp can be traced back to tRNA ancestors.

In **Table 1**, the analysis of substrate and cofactor binding to the ancestral sequences derived from ancestral tRNAs is shown. The data indicate that the ancestral structures have the capacity of binding to simple molecules as magnesium, calcium, ribonucleotides, among others ligands. The most frequent cofactor found in this analysis was magnesium that is a cofactor in the modern RdRp. These analyses indicated that at the origin of the RdRp the most ancient part of the protein is the cofactor-binding site. In modern proteins, the cofactor-binding site is located at the palm domain, which is considered by evolutionary analysis the most ancient domain in the modern RdRp (Poch et al., 1989). These data are in line with the suggestion of Farias et al. (2014, 2016a), where junctions of tRNAs gave origin to the first genes and that RdRp is a very ancient enzyme in the biological systems. The antiqueness of the RdRp reinforces the RNA as the primitive genetic molecule to store the initial biological information. The early emergence of this enzyme in the organization of the biological system played an essential role in the establishment and perpetuation of the first informational modules that worked as the initial metabolic pathways needed for the evolution of the first forms of life.

**Table 1:** The results of the best substrate binding for the models generated by translation of the proto-tRNAs.

tRNA gene ancestors	Coach	TM-site	S-site
tRNA 1	NAD, potassium	Magnesium, potassium, calcium	Magnesium, AMP, calcium
tRNA 2	Calcium	Zinc, calcium	Magnesium, AMP, calcium
tRNA 3	Magnesium	Magnesium, ribonucleic acids, calcium	Chlorophyll, D-manose
tRNA 4	Iodine	Magnesium, zinc, calcium	FAD, zinc, ATP

**FIGURE 3:**
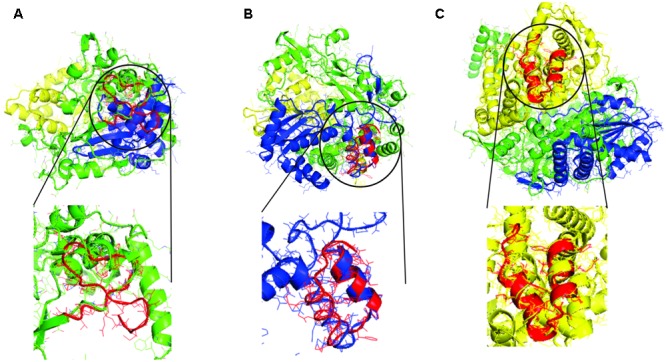
Structural alignments between the models generated by translation of proto-tRNA concatamers and the modern RdRp with the smaller RMSD value. In green the finger domain, in blue the palm domain, in yellow the thumb domain, and in red the structural model generated by translation of proto-tRNA concatamers. **(A)** The structural alignment between tRNA 2 and RdRp of Cardiovirus A, **(B)** the structural alignment between tRNA 3 and RNA Polymerase DSRNA Bacteriophage, and **(C)** the structural alignment between tRNA 3 and RNA polymerase Rotavirus. The structural aligned portion between each tRNA model and the modern protein is highlighted.

It is observed that the tRNA gene ancestors when translated have matches with all modern proteins and with different domains in these proteins. The structural alignment with the smaller RMSD value was between the tRNA 2 translated and the fingers domain of Cardiovirus A with RMSD value of 1.97 (4NZ0); between the tRNA 3 translated and the palm domain of RNA Polymerase DSRNA Bacteriophage with RMSD value of 1.93 (1HHS); and the thumb domain of RNA polymerase Rotavirus with RMSD value of 1.44 (2R7T). In **Figure 3**, the structural alignment that had the smaller RMSD value is illustrated.

## Discussion

**FIGURE 4:**
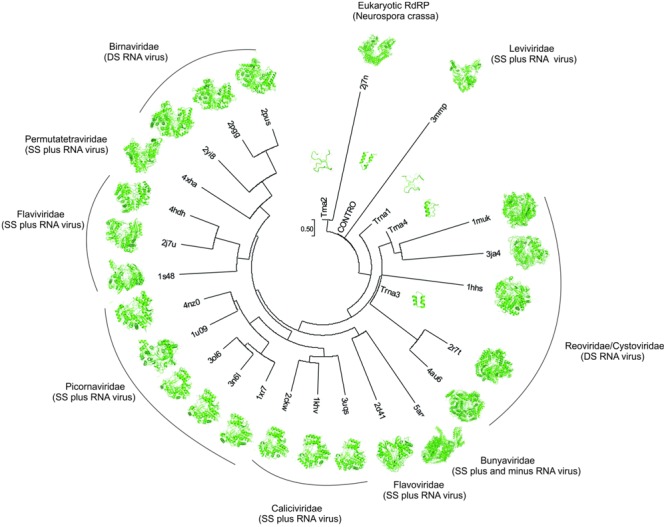
The structural distance phylogenetic tree. tRNA 1, tRNA 2, tRNA 3, tRNA 4, control (contro), RNA Polymerase DSRNA Bacteriophage (PDB: 1HHS); RNA Polymerase Rabbit Hemorrhagic Disease Virus (PDB: 1KHV); RNA Polymerase Sapporo Virus (PDB: 2CKW); Hepatitis C RNA Polymerase (PDB: 2D41); Neurospora Crassa RNA Polymerase (PDB: 2J7N); RNA Polymerase Birnavirus (PDB: 2PGG); RNA Polymerase Infectious Bursal Disease Virus (PDB: 2PUS); RNA Polymerase Rotavirus (PDB: 2R7T); RNA Polymerase Infectious Pancreatic Necrosis Virus (PDB: 2YI8); RNA Polymerase Cypoviruses (PDB: 3JA4); Enterovirus A RNA Polymerase (PDB: 3N6L); RNA Polymerase Norwalk Virus (PDB: 3UQS); RNA Polymerase Rotavirus A (PDB: 4AU6); RNA Polymerase Thosea Assigns Virus (PDB: 4XHA); Rhinovirus A (PDB: 1XR7); Enterovirus C (PDB: 3OL6); Foot-and-Mouth Disease Virus (PDB: 1U09); Cardiovirus A (PDB: 4NZ0); Japanese Encephalitis Virus (PDB: 4HDH); Dengue Virus (PDB: 2J7U); Bovine Viral Diarrhea Virus 1 (PDB: 1S48); Qbeta Virus (PDB: 3MMP); Reovirus (PDB: 1MUK); and La Crosse Bunyavirus (PDB: 5AMQ).

These results suggest that the RdRp originated from junctions of proto-tRNAs that were translated at the emergence of the primitive translation system, when RNA was the informational molecule. It is also suggested that the small domain, which originated from proto-tRNAs, worked as a building block and that the emergence of the fingers and thumb domains occurred by duplication of the first domain, that in turn arose from the palm domain. In **Figure 4**, it is shown the structural tree constructed by Neighbor Joining (NJ). The result indicates that the ancestral structures derived from translated ancestral tRNAs and the control behave like a root, although the tree is not rooted. This condition can reflect the antiqueness of this protein domain. Notice the proximity between the ancestor sequences and the virus family *Leviviridae, Reorividae*, and *Cystoviridae*, which are described as very ancient viral families (Černý et al., 2014). A recent origin for specific RNA viruses, as human hepatitis delta virus (HDV) has been observed (Salehi-Ashtiani et al., 2006). However, the proximity between the RdRp to *Neurospora crassa* and the ancestor RdRp suggests two scenarios. In the first scenario, the ancestor virus lineage was incorporated in the eukaryotic genome and was lost during the evolutionary process, being in this way, an ancient evolutionary event. The second scenario suggests a recent acquisition from a modern virus by lateral gene transfer, and the structural similarity between the eukaryotic RdRp and the ancestor RdRp is a product of evolutionary convergence. In this scenario, the selective pressure that promoted the convergence was the replication of small RNA molecules, both in eukaryotes and in primitive RNA-based genomes.

### Evolution of RNA-Dependent RNA Polymerase and its Implication to RNA Virus Evolution

The evolution of RdRp is intrinsically linked with the evolution of RNA viruses, mainly because this enzyme is responsible for the replication of RNA viral genomes. In **Figure 4**, observe the relationship between the RNA virus families and the evolutionary stages from an ancestor to the modern viruses. This observation is in agreement with other works that traced back the evolutionary history of RNA viruses (Černý et al., 2014; Jácome et al., 2015). An important observation is that the selective pressure under the RdRp is the organization and functioning of the genome, where double-stranded (DS) RNA virus formed a separate group and the single-stranded (SS) RNA virus formed another group. Our results are in line with Černý et al. (2014) and Jácome et al. (2015), which suggested that the transition between SS-RNA and DS-RNA occurred more than once during the evolutionary process. **Figure 4** also suggests that the first viral genome was SS (*Leviviridae*) and evolved to a DS-RNA (*Reoviridae*/*Cystoviridae*). These families of viruses are described as very ancient families (Bamford et al., 2005). This organization of the genome reverts to SS-RNA with the emergence of the families *Flavoviridae, Picornaviridae, Calciviridae, Burnyavoridae*, and *Permutatetraviridae*. A third reversion of the genome organization, from SS-RNA to DS-RNA, occurred with the emergence of *Birnaviridae*, being this condition more recent (Černý et al., 2014). The independence of primers to start the replication can be a primitive characteristic shared between an ancient RdRp and the basal families of RNA viruses. This characteristic gives support to the evolutionary organization observed in **Figure 4**. Evidently, the dependence/independence of a primer emerged more than once during the evolution of RNA viruses (Černý et al., 2014).

The evolutionary position of RNA viruses in the tree of life is a controversial subject in modern biology (Jácome et al., 2015; Nasir and Caetano-Anollés, 2015). Its abundance as parasites of eukaryotic organisms suggests a recent origin, but works that analyzed the evolution of the protein superfamily suggest a very ancient origin, being contemporaries with the last universal common ancestor (Nasir and Caetano-Anollés, 2015). The essentiality of the RdRp in RNA viruses, as well as, in the RNA world, opens questions and scenarios about a recent origin of these viruses. In a RNA world, the emergence of the RdRp is an essential step for the establishment of a primitive genome RNA-based. Thus, the emergence of the RdRp was independent of the viruses and its persistence in cellular genomes made possible the recent origin of RNA viruses, or RNA viruses are ancient and its relation were eliminated in the Archaea domain, nowadays, being in its majority related with bacteria and eukaryotic organisms. Our results suggest an ancient scenario of the origin of RNA viruses, mainly due to the similarities between primitive RdRp derived from tRNA ancestral sequences translated and the modern RNA viruses, as shown in **Figure 4**.

### A Hypothesis for the Evolution of RdRp and the Transition between the RNA World to the DNA/RNA/Protein World

Based in the present results, we suggest a hypothetical scenario where initially a ribozyme with polymerase activity could have enhanced its activity by the binding of a simple cofactor, as magnesium (Shechner et al., 2009; Horning and Joyce, 2017), thus, exerting the functions of replication of some information stored on RNA molecules (Kim and Higgs, 2016; Tagami et al., 2017). With the emergence of the primitive translation system, the first proteins were formed by translation of the junction of proto-tRNAs, and RdRp was among the first enzymes that were formed (Farias et al., 2016b). Accompanied by the compartmentalization process, the control of solutes in the internal environment and the polymerization function by proteins could occur more efficiently than with ribozymes. Initially, the enzyme was formed only by the catalytic loop with capacity of binding to the cofactor and simple molecules as ribonucleotides, that later with duplications and diversifications of the initial catalytic domain, the other parts of the protein emerged. With the establishment of the first domain of RdRp, variants could be generated and mutations could occur, thus, it was possible to generate new proteins, with the conservation of the catalytic site (Zong et al., 2009). With the emergence of a variant with properties of reverse transcriptase, and subsequently a DNA polymerase, a fundamental step occurred to originate the first genomes based in DNA, being the bridge to move from a RNA/Protein World to a DNA/RNA/Protein World (Gilbert, 1986; Müller, 2006). When DNA molecules emerged, by variation, other classes of polymerases appeared. With the complexification of the biological system, functions such as replication, repair, and recombination emerged, very high error rates were selected against, and the new variants arose to work in specific processes.

## Author Contributions

SdF, AdSJ, and MVJ conceived and designed the experiments; SdF, AdSJ, and TGR performed the experiments. SdF, AdSJ, TGR, and MVJ analyzed the data; SdF, TGR, and MVJ contributed to analysis tools; and SdF, AdSJ, and MVJ wrote the paper.

## Conflict of Interest Statement

The authors declare that the research was conducted in the absence of any commercial or financial relationships that could be construed as a potential conflict of interest.

## Footnotes

1 http://zhanglab.ccmb.med.umich.edu/I-TASSER

2 http://zhanglab.ccmb.med.umich.edu/ModRefiner

3 http://zhanglab.ccmb.med.umich.edu/COACH/

4 http://zhanglab.ccmb.med.umich.edu/TM-align/M-align/

5 http://www.trex.uqam.ca
