# Supplementary material for: Origin and Evolution of RNA-Dependent RNA Polymerase
Source: Front Genet. 2017 Sep 20;8:125. doi: 10.3389/fgene.2017.00125 (PMC5611760; doi:10.3389/fgene.2017.00125)
Supplement: Supplementary file 1 [file Data_Sheet_1.DOCX]

Supplementary Material

Origin and evolution of RNA-dependent RNA polymerase

**Savio Torres de Farias^1*^, Ariosvaldo Pereira dos Santos Junior^1^, Thais Gaudêncio Rêgo^2^ and Marco V. José^3*^**

*^1Laboratório de Genética Evolutiva Paulo Leminsk, Departamento de Biologia Molecular, Universidade Federal da Paraíba, João Pessoa, Brazil.^*

*^2Departamento de Informática, Universidade Federal da Paraíba, João Pessoa, Brazil.^*

*^3Theoretical Biology Group, Instituto de Investigaciones Biomédicas, Universidad Nacional Autónoma de México, Ciudad Universitaria, 04510 CDMX, Mexico.^*

*** Correspondence:**Sávio Torres de Farias – [stfarias@yahoo.com.br](mailto:stfarias@yahoo.com.br)

Marco V. José – [marcojose@biomedicas.unam.mx](mailto:marcojose@biomedicas.unam.mx)

# Supplementary Table

| PDB STRUCTURE | FINGERS | PALM | THUMB |
| --- | --- | --- | --- |
| 1HHS | TRNA 2 RMSD= 2.67 | TRNA 1 RMSD= 3.27  TRNA 3 RMSD= 1.93  TRNA 4 RMSD= 2.21 |  |
| 1KHV |  | TRNA 1 RMSD= 2.46  TRNA 2 RMSD= 3.40  TRNA 3 RMSD= 1.96 | TRNA 4 RMSD= 2.99 |
| 1MUK | TRNA 1 RMSD= 3.39 | TRNA 2 RMSD= 3.54 | TRNA 3 RMSD= 2.21  TRNA 4 RMSD= 2.27 |
| 1S48 | TRNA 1 RMSD= 2.80 | TRNA 2 RMSD= 3.48  TRNA 4 RMSD= 3.01 | TRNA 3 RMSD= 1.95 |
| 1U09 | TRNA 1 RMSD= 3,00  TRNA 2 RMSD= 3.67 | TRNA 3 RMSD= 2.15  TRNA 4 RMSD= 3.08 |  |
| 1XR7 |  | TRNA 1 RMSD= 3.40  TRNA 2 RMSD= 3.26  TRNA 4 RMSD= 2.72 | TRNA 3 RMSD=1.97 |
| 2CKW | TRNA 2 RMSD= 3.73 | TRNA 3 RMSD = 2.49 | TRNA 1 RMSD= 2.87  TRNA 4 RMSD= 2.72 |
| 2D41 | TRNA 1 RMSD= 3.49  TRNA 2 RMSD= 3.37  TRNA 3 RMSD= 3.15 | TRNA 4 RMSD= 2.39 |  |
| 2J7N | TRNA 2 RMSD= 2.82 | TRNA 1 RMSD= 3.66 | TRNA 3 RMSD=2.95  TRNA 4 RMSD= 3.29 |
| 2J7U | TRNA 1 RMSD= 3.30  TRNA 4 RMSD= 2.66 | TRNA 3 RMSD= 2.46 | TRNA 2 RMSD= 3,00 |
| 2PGG | TRNA 1 RMSD= 3.26 | TRNA 4 RMSD= 2.51 | TRNA 2 RMSD=3.30  TRNA 3 RMSD=2.59 |
| 2PUS |  | TRNA 1 RMSD= 2.76  TRNA 2 RMSD= 2.30 | TRNA 3 RMSD= 2.84  TRNA 4 RMSD= 2.84 |
| 2R7T | TRNA 2 RMSD= 2.91  TRNA 4 RMSD= 2.49 | TRNA 1 RMSD = 3.45 | TRNA 3 RMSD= 1.44 |
| 2YI8 | TRNA 1 RMSD= 3.33  TRNA 4 RMSD= 3.08 | TRNA 3 RMSD= 3.21 | TRNA 2 RMSD= 2.79 |
| 3JA4 | TRNA 1 RMSD= 3.18  TRNA 3 RMSD= 2.12 | TRNA 2 RMSD= 3.26 | TRNA 4 RMSD= 2.81 |
| 3MMP | TRNA 1 RMSD= 2.70  TRNA 3 RMSD= 2.03 | TRNA 2 RMSD= 3.36  TRNA 4 RMSD= 3.10 |  |
| 3N6L | TRNA 2 RMSD= 4.09 | TRNA 1 RMSD= 3.23  TRNA 3 RMSD= 2.43  TRNA 4 RMSD= 2.78 |  |
| 3OL6 | TRNA 2 RMSD= 4.1 | TRNA 1 RMSD= 3.16  TRNA 4 RMSD= 2.74 | TRNA 3 RMSD= 2.45 |
| 3UQS |  | TRNA 2 RMSD= 3.24  TRNA 3 RMSD= 2.01 | TRNA 1 RMSD= 3.50  TRNA 4 RMSD= 3.14 |
| 4AU6 | TRNA 2 RMSD= 2.35  TRNA 4 RMSD= 2.51 | TRNA 1 RMSD= 3.20 | TRNA 3 RMSD= 1.77 |
| 4HDH | TRNA 1 RMSD= 3.46  TRNA 3 RMSD= 2.28  TRNA 4 RMSD= 2.70 | TRNA 2 RMSD= 3.44 |  |
| 4NZ0 | TRNA 2 RMSD= 1.97 | TRNA 1 RMSD= 2.29  TRNA 3 RMSD= 2.35 | TRNA 4 RMSD= 3.25 |
| 4XHA | TRNA 3 RMSD= 2.05 | TRNA 1 RMSD= 3.12  TRNA 2 RMSD= 2.86  TRNA 4 RMSD= 3.52 |  |
| 5AMQ | TRNA 4 RMSD= 3.11 | TRNA 1 RMSD= 3.23  TRNA 2 RMSD= 3.43  TRNA 3 RMSD= 2.27 |  |

**Table S1**. The RMSD value between the models, the modern proteins and the domain where alignments were observed.
